# Soluble KIT correlates with clinical outcome in patients with metastatic breast cancer treated with sunitinib

**DOI:** 10.1186/1479-5876-10-165

**Published:** 2012-08-16

**Authors:** Kiana Keyvanjah, Samuel E DePrimo, Charles S Harmon, Xin Huang, Kenneth A Kern, William Carley

**Affiliations:** 1 Pfizer Oncology, La Jolla, CA, USA; 2 Previous address: Pfizer Oncology, La Jolla, CA, USA; 3 Current address: Allergan Inc., Irvine, CA, USA; 4 Current address: Johnson & Johnson, San Diego, CA, USA; 5 Current address: Independent consultant, San Diego, CA, USA; 6 Current address: MindPiece Partners, La Jolla, CA, USA

**Keywords:** Sunitinib, Metastatic breast cancer, Biomarkers

## Abstract

**Background:**

Sunitinib inhibits vascular endothelial growth factor receptors (VEGFRs), platelet-derived growth factor receptors, and stem cell factor receptor (KIT). The ability of soluble (s)KIT, VEGF-A, sVEGFR-2, and sVEGFR-3 to predict clinical outcome was analyzed in 61 patients with previously treated metastatic breast cancer (MBC) in a phase II study of sunitinib monotherapy (ClinicalTrials.gov NCT00078000).

**Methods:**

Plasma concentrations of soluble proteins were measured at baseline and during treatment with sunitinib 50 mg/day (4 weeks on treatment, 2 weeks off treatment). Baseline concentrations and maximal percent change during the first two treatment cycles were stratified by median values and evaluated for correlation with median time to tumor progression (TTP) and overall survival (OS). This latter fixed time period was chosen to avoid bias accruing from patients who were on study for longer periods of time.

**Results:**

TTP was significantly longer in patients having median or higher maximal percent sKIT change compared with patients with less than the median change (21.7 vs. 7.9 weeks; p < 0.0001). Similarly, OS was significantly longer in patients having median or higher sKIT change versus less than the median change (53.7 vs. 25.7 weeks; p = 0.018). Significant prolongation of OS (62.6 vs. 32.3 weeks; p = 0.032), but not TTP, was observed in patients with a median or higher maximal percent VEGF-A change compared with less than the median change. Maximal percent change of sVEGFR-2 or sVEGFR-3 concentrations and baseline concentrations of all four proteins were not predictive of clinical outcome.

**Conclusions:**

This exploratory analysis suggests that changes in sKIT and possibly VEGF-A early during sunitinib treatment may be predictive of clinical outcome in MBC.

## Background

Prognostic and predictive biomarkers have long been sought to aid in optimizing therapy and elucidating mechanisms involved in metastatic breast cancer (MBC). An ideal biomarker should be quantifiable early during disease or treatment and be capable of providing evidence for underlying disease mechanisms that may then serve as a therapeutic target. However, the high degree of heterogeneity in MBC has made study of this disease particularly challenging, and, although many biomarkers have been assessed in clinical trials, few have advanced into clinical practice [1].

Sunitinib malate (SUTENT®; Pfizer, Inc.) is an orally administered, small-molecule tyrosine kinase inhibitor with targets that include vascular endothelial growth factor receptor (VEGFR)-1, -2, and -3; platelet-derived growth factor receptor (PDGFR)-α and -β; and stem cell factor receptor (KIT) [2-4]. Sunitinib is approved multinationally for the treatment of metastatic renal cell carcinoma; gastrointestinal stromal tumor (GIST) after disease progression on, or intolerance to, imatinib treatment; and metastatic pancreatic neuroendocrine tumor.

Several pathways inhibited by sunitinib have been implicated in the pathogenesis of breast cancer: for example, expression of VEGF-A, PDGF-AB, and PDGFR-β has been associated with poor prognosis [5-7]. Expression of KIT, a member of the PDGFR subfamily, has also been detected in breast cancer cells with a prevalence of between 1% and 25% [8-14]. A lack of standardized procedures may explain the observed variation in expression of KIT, and while its actual prevalence (and the clinical relevance of its presence in individual breast carcinomas) remains to be determined, it has been suggested that KIT, as part of a broader array of markers, could assist in the appropriate classification of breast cancer patients, and their subsequent assignment to therapy [14]. Elucidation of the pathways responsible for breast cancer would aid identification of patient subpopulations that might benefit from specific targeted therapies.

Results of a previously published phase II trial [15] suggested that single-agent sunitinib had antitumor activity in patients with heavily pretreated MBC (N = 64): an objective response rate (ORR) of 11% was achieved and 5% of patients had stable disease (SD) for ≥ 6 months. Additionally, the ORR in patients with triple-negative tumors (i.e. those negative for estrogen receptor [ER], progesterone receptor, and HER2; n = 20) was 15%.

In this earlier work, a limited analysis of soluble (s) biomarkers (sKIT, VEGF-A, sVEGFR-2, and sVEGFR-3) was undertaken [15]. Results showed that plasma biomarker concentrations changed in response to sunitinib treatment and suggested that these changes correlated with clinical outcomes. The current analysis was undertaken to explore the latter results in more detail using methods distinct from those published previously.

## Methods

### Patients

The study involved female patients ≥ 18 years of age with confirmed breast adenocarcinoma not amenable to surgery, radiation, or curative therapies. Patients had to have had previous treatment with an anthracycline as well as a taxane, and all chemotherapy and radiation treatments must have been completed at least 3 weeks prior to enrollment in the study. Other eligibility criteria were described previously [15].

The study was performed in accordance with the International Conference on Harmonisation Good Clinical Practice guidelines, the Declaration of Helsinki, and applicable local regulatory requirements and laws. The study was approved by the institutional review board or independent ethics committee of each participating center. All patients gave written, informed consent prior to enrollment.

### Study design and treatment administration

This was an open-label, single-arm, phase II study conducted at eight centers in the United States. The primary end point was ORR. Secondary end points included time to tumor progression (TTP), overall survival (OS), safety and tolerability, and an exploration of potential soluble plasma biomarkers of response (sKIT, VEGF-A, sVEGFR-2, and sVEGFR-3). Each treatment cycle consisted of 4 weeks of treatment with sunitinib 50 mg administered orally once daily followed by 2 weeks off treatment (Schedule 4/2) [15].

### Study procedures

Baseline evaluations have been described previously [15]. Response Evaluation Criteria in Solid Tumors were used to determine progression of disease and response to treatment [16]. Plasma samples were taken for analysis of soluble protein biomarkers before sunitinib treatment on study days 1, 14, and 28 of the first treatment cycle, on days 1 and 28 of subsequent cycles, and at the end of treatment. Samples were collected in heparinized tubes. Plasma levels of the biomarkers were analyzed with validated enzyme-linked immunosorbent assay kits or kit components (R&D Systems) in compliance with Good Laboratory Practice guidelines as described previously [17]. Lower limits of quantification for the assays were 31.1, 6.4, 78.1, and 156 pg/mL for sKIT, VEGF-A, sVEGFR-2, and sVEGFR-3, respectively.

### Statistical analysis

Plasma concentrations of soluble proteins were evaluated independently, and descriptive statistics were calculated for each biomarker at each time point. The maximal change in the concentration of each plasma biomarker observed during the first two treatment cycles was calculated for each patient. This fixed time period was chosen to avoid bias accruing from patients who were on study for longer periods of time. Patients who had fewer than two plasma biomarker concentration measurements that were quantifiable within the assay range during the first two treatment cycles (three patients for sKIT, VEGF-A, and sVEGFR-2 measurements and nine patients for sVEGFR-3 measurements, of 64 patients in the study) were therefore excluded from the analysis. The maximal change was calculated as a percentage of the maximal change from cycle 1 day 1 (C1D1) until the end of treatment in cycle 2 (C2D28) as follows [18]:

(1) $$\frac{\left(\text{maximum concentration}–\text{minimum concentration}\right)\times 100}{\text{maximum concentration}}$$

Medians of this value for each protein were used as the cutpoints for stratification of patients into two groups, which were then evaluated for correlation with the efficacy end points TTP and OS. Efficacy comparisons were also carried out in which patients were stratified based on baseline biomarker concentrations, using median values to determine the cutpoints. Time-to-event analyses were performed using the Kaplan-Meier method; results were compared using the Cox proportional hazards model, the Mantel-Haenszel method, and the log-rank test. Univariate and multivariate analyses were performed using the Cox proportional hazards regression model with S+ v8.0 (TIBCO Spotfire). Other analyses were performed using GraphPad Prism 5.1 (GraphPad Software) and Microsoft Office Excel (2003 and 2007).

## Results

### Patient characteristics

In total, 64 patients were enrolled in the study [15]. Patient characteristics at baseline are summarized in Table 1. Tumors were ER-negative in 42% of patients (n = 27), HER2-positive in 19% of patients (n = 12), and triple-negative in 31% of patients (n = 20). All patients had received prior chemotherapy, and all received at least one dose of sunitinib. Details of sunitinib dosing and efficacy and safety outcomes in this study have been reported previously [15].

**Table 1:** Patient demographic and clinical characteristics

**Characteristic**	**Sunitinib (N = 64)**
Median (range) age, years	52 (36–70)
ECOG performance status, n (%)	
0	24 (38)
1	40 (63)
Histologic type, n (%)	
Ductal	54 (84)
Inflammatory	4 (6)
Other	6 (9)
Receptor status, n (%)	
ER-positive	37 (58)
PgR-positive	26 (41)
HER2-positive	12 (19)
Triple-negative	20 (31)
Metastatic sites, n (%)	
Lymph nodes	41 (64)
Liver	38 (59)
Lung	31 (48)
Bone	25 (39)
Pleural effusion	17 (27)
Local recurrence	16 (25)
Skin	14 (22)
Primary tumor	4 (6)
Prior systemic therapy,* n (%)	64 (100)
Anthracycline + taxane + other	60 (94)
Anthracycline + taxane	1 (2)
Anthracycline + other	2 (3)
Other	1 (2)

Adapted from Burstein HJ, et al.: *J Clin Oncol* 2008, **26**(11):1810–1816 [15] with permission. © 2008 American Society of Clinical Oncology. All rights reserved. ECOG, Eastern Cooperative Oncology Group; ER, estrogen receptor; PgR, progesterone receptor. *Eight patients (13%) also received prior trastuzumab treatment.

### Effect of sunitinib treatment on plasma biomarker levels

**Figure 1:**
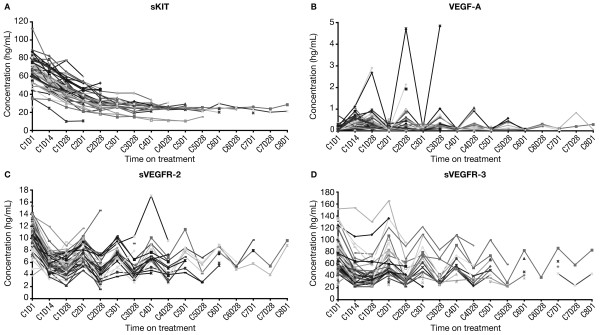
**Plasma protein concentrations in individual patients during treatment with sunitinib.** (**A**) sKIT. (**B**) VEGF-A. (**C**) sVEGFR-2. (D) sVEGFR-3. sKIT, soluble KIT; sVEGFR, soluble vascular endothelial growth factor receptor; VEGF, vascular endothelial growth factor.

As reported previously, significant changes (p < 0.00005) in mean plasma levels were observed for all four biomarkers within the first cycle of sunitinib treatment [15]. Concentrations of sKIT decreased as treatment progressed (for up to eight cycles) irrespective of off-treatment periods (Figure 1A). VEGF-A concentrations generally increased during the 4-week periods on treatment, while sVEGFR-2 and sVEGFR-3 concentrations decreased during the on-treatment periods (Figure 1B-D). Levels of each of these latter three markers returned to near-baseline concentrations at the end of the 2-week off-treatment periods.

**Figure 2:**
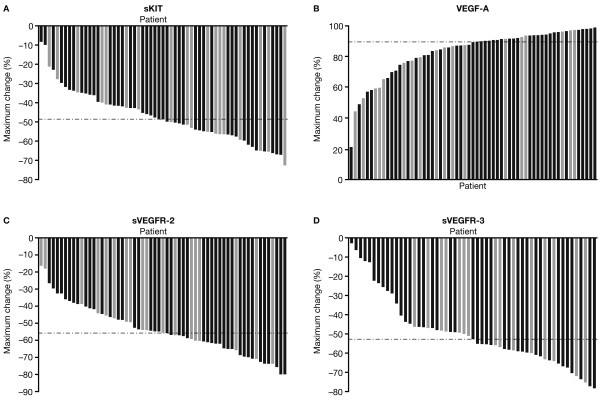
**Maximal percent change in plasma protein concentration from C1D1 to C2D28 by patient.** (**A**) sKIT. (**B**) VEGF-A. (**C**) sVEGFR-2. (**D**) sVEGFR-3. Light gray bars denote patients with triple-negative disease. Broken horizontal lines denote median values for each protein. C, cycle; D, day; sKIT, soluble KIT; sVEGFR, soluble vascular endothelial growth factor receptor; VEGF, vascular endothelial growth factor.

As reported previously, C2D28 was found to be the time point by which mean/median reductions in sKIT levels relative to baseline reached approximately 50%, the cutpoint used in the earlier analysis [15]. C2D28 was also the time point by which the greatest change in plasma concentrations was determined to occur across all four biomarkers by visual inspection of the graphs in Figure 1. Therefore, the median of the maximal percent changes observed from C1D1 to C2D28 was used as the cutpoint for stratification of the study population into two groups (Table 2); these values were 48.6%, 89.4%, 55.8%, and 52.8% for sKIT, VEGF-A, sVEGFR-2, and sVEGFR-3, respectively. Maximal percent changes for individual patients are shown in Figure 2. In patients with triple-negative disease (highlighted in Figure 2), no distinct pattern was apparent with regard to changes in biomarker levels.

**Table 2:** Associations between maximal percent changes in biomarker concentrations or baseline biomarker concentrations and efficacy endpoints

**Biomarker**	**Median value (range)**	**Endpoint**	**< Median value**		**≥ Median value**		HR* (95% CI)	**p**
			**n**	**Median time to event, weeks**	**n**	**Median time to event, weeks**		
**Median maximal percent change in biomarker concentration from C1D1 to C2D28**								
sKIT	48.6% (−)	TTP	30	7.9	31	21.7	5.99 (3.06-11.7)	< 0.0001
		OS		25.7		53.7	2.33 (1.16-4.70)	0.018
VEGF-A	89.4% (−)	TTP	30	9.9	31	10.3	1.75 (0.99-3.09)	0.054
		OS		32.3		62.6	2.12 (1.06-4.23)	0.032
sVEGFR-2	55.8% (−)	TTP	30	10.1	31	10.2	1.07 (0.62-1.85)	0.81
		OS		36.0		53.7	1.52 (0.76-3.02)	0.24
sVEGFR-3	52.8% (−)	TTP	27	10.1	28	11.0	1.40 (0.77-2.52)	0.27
		OS		51.6		52.7	1.98 (0.79-4.96)	0.15
**Median baseline biomarker concentration**								
sKIT	70 ng/mL (26–113)	TTP	28	10.6	28	10.1	1.08 (0.60-1.96)	0.80
		OS		37.4		45.1	1.13 (0.55-2.35)	0.74
VEGF-A	53 pg/mL (14–709)	TTP	25	10.1	26	10.2	0.71 (0.38-1.33)	0.29
		OS		Not reached		33.0	0.55 (0.25-1.22)	0.14
sVEGFR-2	11 ng/mL (4–15)	TTP	28	10.3	28	10.1	0.98 (0.54-1.76)	0.94
		OS		37.4		62.6	1.19 (0.57-2.46)	0.65
sVEGFR-3	68 ng/mL (25–152)	TTP	28	10.1	28	10.2	0.97 (0.54-1.75)	0.93
		OS		37.4		53.7	1.43 (0.68-2.98)	0.35

C, cycle; CI, confidence interval; D, day; HR, hazard ratio; OS, overall survival; sKIT, soluble KIT; sVEGFR, soluble vascular endothelial growth factor receptor; TTP, time to tumor progression; VEGF, vascular endothelial growth factor.

*HR > 1 favors the groups with values ≥ median value.

### Correlation between early changes in plasma biomarker levels and clinical outcome

**Figure 3:**
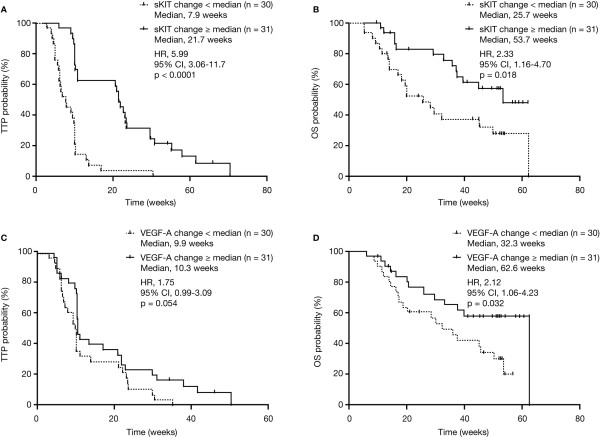
**TTP and OS by median maximal percent change in plasma protein concentration through C2D28.** (**A**) TTP by sKIT concentration. (**B**) OS by sKIT concentration. (**C**) TTP by VEGF-A concentration. (**D**) OS by VEGF-A concentration. C, cycle; D, day; OS, overall survival; sKIT, soluble KIT; TTP, time to tumor progression; VEGF, vascular endothelial growth factor.

Sixty-one patients had two or more plasma sKIT measurements during the first two treatment cycles. Median TTP was 21.7 weeks in patients having a maximal percent sKIT change equivalent to the median or higher (n = 31) compared with a median TTP of 7.9 weeks in patients with less than the median maximal sKIT change (n = 30; hazard ratio [HR], 5.99; 95% confidence interval [CI], 3.06-11.7; p < 0.0001; Table 2; Figure 3A). Patients with sKIT changes equivalent to the median or higher had a median OS of 53.7 weeks compared with 25.7 weeks in patients with less than the median change (HR, 2.33; 95% CI, 1.16-4.70; p = 0.018; Table 2; Figure 3B).

Sixty-one patients had two or more plasma VEGF-A measurements during the first two treatment cycles. Patients with a maximal percent VEGF-A change equivalent to the median or higher (n = 31) had a median TTP of 10.3 weeks compared with 9.9 weeks in patients with less than the median change in VEGF-A (n = 30; HR, 1.75; 95% CI, 0.99-3.09; p = 0.054; Table 2; Figure 3C). Similarly, in the group with a VEGF-A change equivalent to the median or higher, patients had a median OS of 62.6 weeks compared with 32.3 weeks in the patients with less than the median change in VEGF-A (HR, 2.12; 95% CI, 1.06-4.23; p = 0.032; Table 2; Figure 3D).

No statistically significant differences in TTP or OS were observed between groups when patients were stratified based on the maximal percent change in plasma sVEGFR-2 or sVEGFR-3 concentrations (Table 2).

In addition to changes in plasma biomarker concentrations, we evaluated the relationships between baseline demographic and disease characteristics (including age, race, hormone status, and Eastern Cooperative Oncology Group performance status) and clinical outcomes in univariate and multivariate analyses (Table 3). Among these, a significant association with TTP was only found for race. In multivariate analysis using models combining sKIT change and race with or without VEGF-A change, greater sKIT change (evaluated either as a categorical or a continuous variable) was highly predictive of improved TTP (p < 0.0001), as was non-white race (p = 0.0055), while VEGF-A change (categorical variable) did not show a statistically significant association. In univariate analysis of OS, only age showed a significant correlation among baseline characteristics and was included in multivariate models with sKIT with or without VEGF-A change. Greater sKIT change (either as a categorical or continuous variable) and age < 65 (noting that this group represented 98% of patients) were significantly associated with improved OS in the multivariate analyses (p = 0.0085, < 0.0001, and = 0.0045, respectively), while greater VEGF-A change (categorical variable) and age evaluated as a continuous variable showed marginally significant associations (both p = 0.047).

**Table 3:** Univariate and multivariate analysis of relationships between baseline patient characteristics or early changes in biomarker concentrations and clinical outcome

	n*	**TTP**			**OS**		
		**HR**^**†**^	**95% CI**	**p**	**HR**^**†**^	**95% CI**	**p**
**Univariate analysis**							
Age (< 65 vs. ≥ 65 years)	59/5	1.19	0.43-3.33	0.74	3.09	1.17-8.16	0.023
Race (white vs. non-white)	52/11	0.39	0.18-0.86	0.019	0.67	0.26-1.72	0.40
ECOG performance status (0 vs. ≥ 1)	24/40	1.20	0.70-2.04	0.53	1.58	0.78-3.21	0.21
ER (+ vs. −)	37/27	1.14	0.66-1.96	0.64	1.60	0.80-3.20	0.18
PgR (+ vs. −)	26/35	0.88	0.50-1.54	0.65	1.60	0.81-3.16	0.17
HER2 (+ vs. −)	12/48	0.74	0.38-1.45	0.38	0.70	0.29-1.69	0.43
Triple-negative (yes vs. no)	20/43	0.99	0.56-1.76	0.97	0.67	0.31-1.42	0.29
Baseline sKIT (continuous^‡^)	61	1.00	0.97-1.02	0.82	0.99	0.96-1.02	0.48
Baseline VEGF-A (continuous^‡^)	63	1.11	0.98-1.27	0.11	1.10	0.95-1.28	0.22
Baseline sVEGFR-2 (continuous^‡^)	62	0.99	0.89-1.10	0.83	0.91	0.80-1.03	0.15
Baseline sVEGFR-3 (continuous^‡^)	54	1.00	0.99-1.01	0.77	1.00	0.99-1.01	0.97
sKIT change (≥ 48.6% vs. < 48.6%)	30/31	4.36	2.38-7.97	< 0.0001	2.28	1.13-4.60	0.022
VEGF-A change (≥ 89.4% vs. < 89.4%)	31/30	1.71	0.99-2.96	0.054	2.25	1.09-4.65	0.029
sVEGFR-2 change (≥ 55.8% vs. < 55.8%)	31/30	1.14	0.67-1.94	0.63	1.37	0.68-2.74	0.37
sVEGFR-3 change (≥ 52.8% vs. < 52.8%)	28/27	1.39	0.79-2.45	0.26	1.66	0.79-3.52	0.18
sKIT change (continuous^‡^)	61	0.93	0.91-0.96	< 0.0001	0.94	0.92-0.97	< 0.0001
VEGF-A change (continuous^‡^)	61	0.99	0.97-1.01	0.21	0.98	0.96-1.00	0.042
sVEGFR-2 change (continuous^‡^)	61	1.00	0.98-1.02	0.95	0.99	0.97-1.01	0.45
sVEGFR-3 change (continuous^‡^)	55	0.99	0.98-1.01	0.38	0.98	0.96-1.00	0.051
**Multivariate analysis of factors showing significance in univariate analysis**^**§**^							
Model using categorical variables							
Age (< 65 vs. ≥ 65 years)	−	−	−	−	4.45	1.59-12.48	0.0045
Race (white vs. non-white)	−	0.30	0.12-0.70	0.0055	−	−	−
sKIT change (≥ 48.6% vs. < 48.6%)	−	4.75	2.48-9.08	< 0.0001	2.69	1.29-5.64	0.0085
VEGF-A change (≥ 89.4% vs. < 89.4%)	−	1.62	0.93-2.80	0.086	2.10	1.01-4.34	0.047
Model using continuous variables							
Age (continuous^‡^)	−	−	−	−	1.05	1.00-1.09	0.047
Race (white vs. non-white)	−	0.25	0.11-0.61	0.0021	−	−	−
sKIT change (continuous^‡^)	−	0.92	0.90-0.95	< 0.0001	0.94	0.92-0.97	< 0.0001

CI, confidence interval; ECOG, Eastern Cooperative Oncology Group; ER, estrogen receptor; HR, hazard ratio; OS, overall survival; PgR, progesterone receptor; sKIT, soluble KIT; sVEGFR, soluble vascular endothelial growth factor receptor; TTP, time to tumor progression; VEGF, vascular endothelial growth factor.

*Number of patients analyzed in univariate analysis (for categorical variables, numbers for both groups are shown).

^†^For categorical variables, HR > 1 favors the first category and HR < 1 favors the second category; for continuous variables, HR > 1 favors the value when it decreases and HR < 1 favors the value when it increases.

^‡^Modeled as a continuous variable.

^*§*^n = 60 for the TTP models because race was unknown in one patient, and n = 61 for the OS models.

### Correlation between baseline plasma biomarker levels and clinical outcome

When patients were stratified based on median baseline concentrations, no statistically significant differences in TTP or OS were detected between the groups of patients with baseline concentrations above or below the median values (Table 2).

## Discussion

We performed a detailed analysis of the ability of four plasma proteins (sKIT, VEGF-A, sVEGFR-2, and sVEGFR-3) to predict clinical outcome with sunitinib in patients with previously treated MBC. Biomarker cutpoints were assessed using two different parameters: percent maximal change in biomarker concentration during the first two treatment cycles and median baseline concentrations. Among these analyses, changes in the levels of sKIT during treatment showed the strongest associations with clinical outcome, with greater reductions in sKIT levels being predictive of improved TTP and OS. Notably, of the seven patients in the study who had confirmed partial responses, six had changes in sKIT levels that were greater than or equal to the median value (the seventh had fewer than two quantifiable plasma samples within the first two treatment cycles and was unevaluable for this analysis; data not shown). Greater increases in VEGF-A levels showed a trend towards an association with improved TTP and a statistically significant association with improved OS. These results were confirmed in multivariate analyses, in which changes in sKIT levels were shown to be a statistically significant predictor of both TTP and OS, and VEGF-A change, a marginally significant predictor of OS. Baseline concentrations of all four proteins using median values to determine cutpoints were not found to be predictive of clinical outcome.

Determination of appropriate cutpoints for stratifying patients in exploratory biomarker analyses is often empirical and arbitrary in nature. Previous analyses of the current dataset involved normalization of biomarker data at each time point relative to its value at baseline, and although statistically significant correlations were also shown between decreases in plasma concentrations of sKIT (decreases by ≥ 50% at the start or end of the last treatment cycle relative to baseline) and improved clinical outcomes [15], the methodology employed assumed that intrapatient variability was low, and that the standard errors between samples taken at different time points were similar. The use of percent maximal change in biomarker concentrations in the current analysis has the advantage of effectively circumventing assumptions about intrapatient variability [18].

The results obtained in the present analysis, together with those obtained from earlier analyses of the same dataset [15], are suggestive of early changes in sKIT levels being a biomarker of clinical outcome with sunitinib in MBC. However, in breast cancer, the role of KIT remains unclear. Studies comparing levels of both KIT mRNA and protein with breast tumors and normal breast tissue have yielded conflicting results (reviewed in reference [19]). A more recent report, however, examining a large series of breast carcinomas (n = 924), concluded that KIT was expressed in 15% of breast cancer patients and was a prognostic indicator of poor clinical outcome [14]. Activating *KIT* mutations have not so far been reported in breast cancer, although research to date has utilized patient series of limited size [10]. Moreover, clinical results obtained with sunitinib in phase III studies and with imatinib (also a KIT inhibitor) in phase II studies utilizing broad populations of patients with HER2-negative advanced breast cancer (without selection for any biomarker), whether alone or in combination with cytotoxic chemotherapies, have been disappointing [20-26]. However, all of these sunitinib studies in advanced breast cancer utilized sunitinib at 37.5 mg on a continuous daily dosing (CDD) schedule, rather than 50 mg/day on Schedule 4/2 as in the present study. Additionally, single-agent sunitinib (at 37.5 mg on the CDD schedule) was recently found to be less effective than standard-of-care chemotherapies in patients with previously treated advanced triple-negative breast cancer (TNBC) [27], a type of breast cancer thought to be associated with higher frequencies of KIT expression [9,28,29]. Although partial responses were observed in three of 20 (15%) patients with tumors that were triple-negative in the present study [15], a clear correlation did not appear to exist between sKIT changes and TNBC *per se* (Figure 2A). KIT overexpression has also been reported to occur more frequently in ductal carcinomas compared with other breast cancer histologies [11]. While all tumor responses in the present study occurred in patients whose tumors had a ductal histology, the tumors of most patients in the study overall were classified as ductal (84%) [15], precluding any conclusions being drawn about relationships between histologic type and the effects of sunitinib on the biomarkers measured. Given the heterogeneity of breast cancer overall and of sub-types such as TNBC in particular, a prospective biomarker-driven study would be required to definitely assess the role of KIT and the utility of sKIT in determining the outcomes of breast cancer patients treated with KIT inhibitors.

In contrast to the results obtained with sKIT changes during sunitinib treatment, baseline sKIT concentrations were not found to be predictive of TTP or OS in our analyses. A similar lack of correlation between baseline sKIT levels and clinical outcomes was reported in GIST [30], a tumor type in which activating mutations yielding constitutively active KIT proteins occur in approximately 80% of tumors [31]. In clinical trials of patients with GIST, treatment with either sunitinib (both at 50 mg/day on Schedule 4/2 and at 37.5 mg on the CDD schedule) or imatinib was found to be highly efficacious, suggesting that KIT inhibition was critical for tumor control [32-34]. In addition, a decline in plasma sKIT levels after two cycles of sunitinib treatment has been shown to function as a potential surrogate marker for TTP in GIST [30]. That report noted that a relatively large component of physiologic sKIT is likely to be unrelated to the tumor at baseline, given the levels of sKIT found in healthy individuals [35].

The limited associations seen in the present analyses between greater changes in VEGF-A levels and improved outcomes were not noted in the earlier analysis of this dataset [15]. However, with increases in circulating VEGF-A being a well-known pharmacodynamic effect of sunitinib treatment [15,17], an association between greater pharmacodynamic changes and improved outcomes is not unexpected. Such a potentially predictive association has also been reported in sunitinib-treated renal cell carcinoma [17,36] and hepatocellular carcinoma [37], but not GIST [38,39]. Possible associations between VEGF-A levels and clinical outcomes have also been evaluated with other antiangiogenic agents. For example, baseline levels of plasma VEGF-A were recently reported to be a potential biomarker of improved clinical outcomes with the anti-VEGF monoclonal antibody bevacizumab in patients with advanced gastric cancer [40]. Additionally, high baseline levels of VEGF-A have been associated with poor prognosis in breast cancer and other tumor types [5,41]. In our analyses, it may be worth noting that there was a trend towards an association between low baseline VEGF-A levels and improved outcomes (Tables 2 and 3), although this did not reach statistical significance.

The earlier analysis of this dataset noted a trend towards an association between decreases of sVEGFR-3 levels of ≥20% at the start of cycle 2 (or the last treatment cycle) and longer OS (p = 0.07) [15]. In the present analysis, no association between changes in sVEGFR-3 levels and clinical outcome was observed.

The current analyses were limited by several factors. One such limitation was their retrospective nature, which restricts clinical interpretation of the data. Small sample size was another limitation: as in many clinical trials, the study was powered to support the primary endpoint (ORR), with biomarkers evaluated only as a secondary objective. Additionally, the study utilized a relatively unselected patient population as described previously. Finally, because this was a single-arm, non-comparative study, it was unable to distinguish whether any biomarkers identified were prognostic or predictive in nature.

## Conclusions

The current exploratory analysis suggests that changes in sKIT and possibly VEGF-A observed during early treatment cycles may serve as predictive markers for clinical outcome (TTP and OS) with sunitinib in this patient population. The inability to consistently demonstrate broad clinical benefit with sunitinib and other targeted agents in HER2-negative, but otherwise unselected, populations of patients with advanced breast cancer in recently reported phase III studies highlights the urgent need to identify biomarkers of efficacy to identify specific subsets of patients that do benefit. The associations between changes in sKIT and clinical outcome described in this study suggest that better characterization of breast cancer subtypes expressing KIT and elucidation of its role in control of tumor growth may be worthwhile. Our results and methodology may also be applicable to the development of other KIT inhibitors in this heterogeneous patient population.

## Abbreviations

C, Cycle; CDD, Continuous daily dosing; CI, Confidence interval; D, Day; ER, Estrogen receptor; GIST, Gastrointestinal stromal tumor; HER2, Human epidermal growth factor receptor 2; HR, Hazard ratio; KIT, Stem cell factor receptor; MBC, Metastatic breast cancer; ORR, Objective response rate; OS, Overall survival; PDGF, Platelet-derived growth factor; PDGFR, PDGF receptor; Schedule 4/2, 4 weeks on treatment followed by 2 weeks off treatment; SD, Stable disease; sKIT, Soluble KIT; sVEGFR, Soluble VEGFR; TNBC, Triple-negative breast cancer; TTP, Time to tumor progression; VEGF, vascular endothelial growth factor; VEGFR, VEGF receptor.

## Competing interests

All of the authors are or were employees of Pfizer and all except Kiana Keyvanjah hold or held Pfizer stock.

## Authors’ contributions

SD and XH were involved in the conception and design of the study. SD was involved in acquisition of the biomarker data. WC and KK helped to draft the manuscript. All authors analyzed and interpreted the data and revised the manuscript for important intellectual content. All authors read and approved the final manuscript.
